# Lichtenstein and Total Extraperitoneal Techniques in Inguinal Hernia Surgery: A Comparison of the Intraoperative and Early Postoperative Complications Between the Two Approaches

**DOI:** 10.7759/cureus.28020

**Published:** 2022-08-15

**Authors:** Abdullah Yıldız

**Affiliations:** 1 General Surgery Department, University of Health Sciences Ümraniye Training and Research Hospital, Istanbul, TUR

**Keywords:** laparoscopic tep, tep approach, total extraperitoneal techniques, lichtenstein techniques, totally extraperitoneal, open inguinal hernia, laparoscopy, inguinal hernia

## Abstract

Background and objective

The Lichtenstein technique (LSt) and total extraperitoneal technique (TEPt) are the most frequently performed surgical procedures for inguinal hernia (IH). This study aimed to compare LSt and TEPt in terms of intraoperative and postoperative complications as well as recurrence rates.

Methods

This retrospective study involved patients hospitalized for IH repair. The study included a total of 262 patients (LSt group: n=125; TEPt group: n=137).

Results

The follow-up period of the patients ranged between 16 and 30 months (mean: 22.3 months). While intraoperative complications were more commonly reported in the TEPt group, postoperative complications were more common in the LSt group (2.9% vs. 1.6%). Postoperative bleeding/hematoma developed in three (2.4%) patients in the LSt and three (2.2%) in the TEPt group. One of the patients in the LSt group was reoperated due to persistent bleeding. Two patients in the LSt and two in the TEPt group were reoperated for postoperative recurrence (1.6% vs. 2.4%). One patient was reoperated due to chronic persistent pain. Seroma was aspirated in three (2.4%) patients in the LSt and two (1.5%) in the TEPt group.

Conclusion

This study revealed no significant difference between TEPt and LSt groups regarding intraoperative and postoperative complications. We propose that both TEPt and LSt could be safely performed in cases of primary and complex IH by selecting the appropriate method based on the hernia type and patient and with sufficient surgical experience.

## Introduction

The field of inguinal hernia (IH) surgery has undergone remarkable changes in the past few decades. Thanks to the use of mesh in hernia surgery, significant reductions in recurrence rates have been achieved [1]. Tension-free mesh repair has led to a new revolution in the surgical community [2]. The Lichtenstein technique (LSt) is one of the most preferred open procedures in IH surgery. Laparo-endoscopic methods, since they are less painful, and lead to faster recovery, early return to daily activities, and better cosmetic outcomes, have cemented their place in hernia surgery as popular modalities [3,4].

Based on the Swedish Hernia Registry, LSt was performed in 64% of patients and totally extraperitoneal technique (TEPt) in 25% until 2015 [5]. The German Herniamed Registry system has reported that transabdominal preperitoneal technique (TAPPt) has been performed in 39%, TEPt in 25%, and LSt in 24% of patients from 2009 to 2016 [6].

The European Hernia Society (EHS), the International Endohernia Society (IEHS), and the European Association for Endoscopic Surgery (EAES) have published a consensus document focusing on laparo-endoscopic treatments. These three associations began to cooperate in 2014, concluding that it was essential to establish universal guidelines for treating IH. In the guidelines published by the IH study groups, criteria depending on the evidence level have been presented for which type of hernia treatment to be applied (open or laparoscopic techniques). They also reported that the choice of surgery varies depending on the person, hernia type, and the surgeon's experience [1,4,6]. The outcomes have been subject to vigorous debates due to severe complications in recent years, especially in the early years of applying laparo-endoscopic techniques [1,6,7]. However, it is essential to note that the laparoscopic TEPt could be successfully performed in primary and large inguinoscrotal and complex hernias by surgeons with sufficient and relevant laparoscopic surgery experience. The most common problems experienced by surgeons in the laparoscopic TEPt pertain to challenges in managing issues such as intraoperative bleeding, pneumoperitoneum (PP) due to peritoneal rupture, and conversions [8,9,10].

This study aimed to compare LSt and laparoscopic TEPt in terms of intraoperative and postoperative results such as vascular and visceral injuries, hematoma, seroma, chronic pain, and recurrence rates.

## Materials and methods

This retrospective study involved patients who presented to the Health Sciences University, Ümraniye Training and Research Hospital for IH repair between October 2015 and December 2020. Intraoperative and postoperative data were recorded prospectively (including video recordings). This study included 262 patients (LSt group: n=125; TEPt group: n=137). All procedures (LSt, TEPt) and data analyses were performed by a single surgeon with experience in advanced laparoscopic surgery. The Ethics Committee's approval was obtained (Apr 14, 2020/82). Informed consent was not obtained from the patients since this study was designed as a retrospective analysis. Besides, the identity and health information of the patients were protected in line with the ethical principles of the Declaration of Helsinki, ensuring confidentiality and privacy.

The inclusion criteria were as follows: patients with elective primary, unilateral, bilateral, femoral, and complex hernias (recurrent, chronic incarcerated, or irreducible hernias, scrotal hernias), those who were 18-80 years of age, and eligible for regional or general anesthesia.

Patients at high risk of postoperative complications due to uncontrolled comorbidities, diabetes, concomitant malignancy, or immunodeficiency, those with an American Society of Anesthesiologists (ASA) score of 4, those who were pregnant, and patients with coagulation disorders, as well as emergency cases were excluded from the study.

Lichtenstein technique

The surgery was performed under spinal or general anesthesia. An oblique incision of about 4-6 cm was made on the hernia side. The ilioinguinal and iliohypogastric nerves were carefully identified and preserved at this stage. Explorations were carefully performed for hernias (lateral, median, and femoral). After the hernia sacs were prepared, they were reduced or resected according to the type and structure of the hernia. A polypropylene mesh of 7 x 10-12 cm was shaped and fixed with non-absorbable polypropylene sutures.

Total extraperitoneal technique

All laparoscopic cases were performed under general anesthesia. A 10-12-mm incision was made just below the umbilicus. Insufflation was achieved by placing a 12-mm trocar in front of the posterior rectus fascia towards the preperitoneal space. The gas pressure was set to 12 mmHg. The preperitoneal area was prepared using a balloon dissector or a camera for dissection. The screen was set so that it was on the side of the hernia and the opposite side of the operator. The inferior epigastric vessels, pubic tubercle, and Cooper's ligament must be seen at this stage. Two additional 5-mm trocars were placed between the umbilicus and the pubic tubercle. Careful dissection is essential to avoid bleeding and peritoneal perforation. The lateral (inferior epigastric vessels), medial (umbilical artery), median umbilical (urachus) folds, pubic tubercle, and Cooper's ligament were identified for reliable dissection.

After preparing the hernia sac, the Bogros space was designed by parietalization of the cord to insert the mesh. At this stage, nerves in the triangle of pain were preserved. In large direct hernias, it was fixed to the posterior inguinal wall with sutures from the base of the sac to Cooper's ligament or over the iliopubic tract in some cases. The sizeable laying mesh of 15 x 10-12 cm covered all Myopectineal orifices and was fixed with absorbable tackers.

Data collection

Demographic data, preoperative and intraoperative variables, duration of procedures, complications, and length of hospital stay were recorded. The postoperative data of all patients were collected and analyzed based on the hospital registry system, telephone interviews, or invitations to the hospital.

Statistical analysis

The SPSS Statistics version 15.0 (IBM Corp., Armonk, NY) for Windows was used for statistical analysis. Descriptive statistics - numbers and percentages for categorical and numerical variables - were presented in averages, standard deviations, and ranges. The ratios were compared with the square test in the groups. Since the numerical variables met the normal distribution condition, the comparisons of the two independent groups were made with the student's t-test. A p-value <0.05 was considered statistically significant.

## Results

The follow-up period of the patients ranged between 16 and 30 months (mean: 22.3 months). Only 29% of the patients in the LSt group were operated on under spinal anesthesia, while others were given general anesthesia. In contrast, all patients in the TEPt group were operated on under general anesthesia. The mean duration of operations for the LSt and TEPt groups was 37.51 and 44.33 minutes, respectively. The difference in age, ASA score, and administered anesthesia between the groups were statistically significant (p<0.001) (Table 1).

**Table 1:** Demographic and clinical characteristics of the patients ASA: American Society of Anesthesiologists; LSt: Lichtenstein technique; SD: standard deviation; TEPt: total extraperitoneal technique

Characteristics		LSt group	TEPt grup	P-value
Number of patients		125	137	
Gender, n (%)	Male	108 (86.4%)	121 (88.3%)	0.640
	Female	17 (13.6%)	16 (11.7%)	
Age, years, mean ±SD (min-max)		53.2 ±14.0 (20-80)	43.9 ±11.1 (19-70)	<0.001
Anesthesia, n (%)	General	66 (71.0%)	137 (100%)	<0.001
	Spinal	27 (29.0%)	0 (0.0%)	
ASA classification, n (%)				
I		65 (68.4%)	112 (81.8%)	0.005
II		25 (26.3%)	25 (18.2%)	
III, IV		5 (5.3%)	0 (0.0%)	
Operation time, minutes, mean ±SD		37.51 ±14.04	44.3 ±11.2	0.710
Hospital stay, days, mean ±SD		1.3 ±0.7	1.2 ±0.3	0.600
Body mass index, kg/m^2^,mean ±SD		23.1 ±3.6	24.7.0	0.180
Chronıc irreducıble cases, n (%)		7 (5.6%)	3 (2.2%)	0.201
History of abdominopelvic surgery, n (%)		5 (4.0%)	8 (5.8%)	0.493
Recurrent (preoperatively) cases, n (%)		6 (4.8%)	4 (2.9%)	0.526

When analyzing the groups regarding laterality hernia characteristics, there were seven (5.6%) bilateral hernia cases in the LSt group and 44 (32.1%) in the TEPt group (p<0.001) (Table 2). Polypropylene mesh was routinely used in all our patients.

**Table 2:** Distribution of the patients according to laterality and types of inguinal hernia LSt: Lichtenstein technique; TEPt: total extraperitoneal technique

Varıables	LSt group, n (%) (n=125)	TEPt group, n (%) (n=137)	P-value
Laterality			
Right	72 (57.6%)	46 (33.6%)	<0.001
Left	46 (36.8%)	36 (26.3%)	0.067
Bilateral	7 (5.6%)	44 (32.1%)	<0.001
Hernia type			
Medial	49 (39.2%)	38 (27.7%)	0.049
Lateral	76 (60.8%)	72 (52.6%)	0.179
Femoral	4 (3.2%)	4 (2.9%)	1.000
Scrotal descent	15 (12.0%)	13 (9.5%)	0.511

Although there was no significant difference between the groups regarding the total complication rate, it was higher in the LSt group (p=0.979) (Table 3).

Perioperative bleeding is usually caused by an injury to the epigastric or spermatic vessels during trocar insertion or hernial sac dissection. Bleeding was reported in two patients in the LSt group and four in the TEPt group. The study revealed that conversion occurred in four (2.9%) TEPt cases. Out of eight (5.8%) cases in which PP developed, three (2.2%) were converted to open technique because of the restriction of the working area due to PP caused by a peritoneal tear during hernia sac dissection. One case was caused by sigmoid colon injury. The patient who developed sigmoid colon perforation had a left recurrent IH history. The injury occurred during the dissection of the hernial sac (sliding hernia; the contents of the sac were sigmoid colon). In this case, the surgery was performed using both the open and TAPP procedure (hybrid method).

Hematoma developed in three patients in the LSt group, and one of these patients was reoperated on the first postoperative day due to early bleeding. Other hematomas healed spontaneously in four and six weeks on average.

Bleeding/hematoma developed in the left Bogros space in the early postoperative period in a patient who was operated on with the diagnosis of bilateral IH. This patient had a history of left varicocelectomy. Since the bleeding persisted despite two units of blood transfusion, angiography was performed. The patient was diagnosed with a false aneurysm and was treated with endovascular embolization. It was reported that the bleeding was caused by one of the terminal branches of the left internal iliac artery. Although not statistically significant, symptomatic and asymptomatic seroma were more common in LSt repair than in TEPt. Symptomatic seroma was aspirated in three patients in the LSt group and two in the TEPt group (Table 3).

Chronic persistent pain was reported in four patients in the LSt and two in the TEPt group. Only one patient who underwent LSt was reoperated after surgery for chronic persistent pain. Mesh extraction and ilioinguinal neurectomy were performed on this patient because the mesh was massively adherent to the nerve. A patient with a preoperative diagnosis of recurrent scrotal hernia underwent an orchiectomy in the same session. Atrophic testis had been previously observed in this patient on physical examination and scrotal ultrasonography (USG) (consent had been obtained for possible orchiectomy preoperatively). Nine patients in the TEPt group developed a peritoneal tear, including two with a history of abdominopelvic surgery. Conversion of TEPt to LSt occurred only in four (2.9%) of these cases (Table 3).

Chronic wound infection was not observed in the groups, but superficial wound infection was reported in the LSt group (5.37%). Cord edema and local stiffness were more common in the LSt group, although the difference between the two groups was not significant. Mesh rejection or testicular complications were not observed in any patient in the series. At a mean follow-up of two years, approximately 2% (1.6%) of the patients in the LSt group and 2% (1.5%) in the TEPt group were operated on for recurrence (Table 3).

**Table 3:** Distribution of complications in the patients LSt: Lichtenstein technique; TEPt: total extraperitoneal technique

Variables	LSt group, n (%) (n=125)	TEPt group, n (%) (n=137)	P-value
Total complications	13 (10.4%)	12 (8.7%)	0.979
Intraoperative complications			
Vascular injury	2 (1.6%)	4 (2.9%)	0.686
Visceral injury	0 (0.0%)	1 (0.7%)	1.000
Postoperative complications			
Hematoma	3 (2.4%)	3 (2.2%)	1.000
Seroma requiring aspiration	3 (2.4%)	2 (1.5%)	0.672
Asymptomatic seroma	12 (9.6%)	9 (6.6%)	0.367
Early reoperation	1 (0.8%)	0 (0.0%)	0.373
Chronic persistent pain	4 (3.2%)	2 (1.5%)	0.429
Conversion	0 (0.0%)	4 (2.9%)	0.124
Cord edema	13 (10.4%)	9 (6.6%)	0.264
Recurrences (reoperated)	2 (1.6%)	2 (1.5%)	1.000
Mortality	0 (0.0%)	0 (0.0%)	-

## Discussion

The inguinal canal anatomy is complex and includes vital anatomical structures for successful surgical outcomes; anatomical knowledge, appropriate technique, appropriate patient selection, adequate technical equipment, and sufficient surgical experience are essential to overcome the risks of complications or challenges. The best surgical approach should be easy to learn, have a low risk of complications, lead to rapid recovery, have acceptable recurrence rates, and be cost-effective. In IH surgery, obese patients, preoperative recurrences, history of pelvic surgery, and complex (large scrotal, chronic incarcerated, or unreduced) hernias are the most challenging cases for surgeons. Today, laparo-endoscopic techniques have become an alternative to the LSt in IH surgery, thanks to the comfort they provide to the patient [1].

Laparoscopic TEPt is generally recommended as a viable procedure for primary unilateral, bilateral, and occult or contralateral herniations [10]. However, the inability to control the sac content in cases of chronic incarcerated or uneducable hernia is considered a disadvantage for TEPt [4,5]. When LSt and TEPt were evaluated regarding total complications, intraoperative complications were reported more in the TEPt procedure in some studies (p=0.0359) while postoperative complications were reported more in the LSt procedure (p=0.001) [5]. However, this study found no difference between the groups regarding total complications.

The field-of-view or working-space restrictions due to bleeding and PP caused by peritoneal tear are the most important causes of conversion in TEPt [11]. In this study, bleeding from the lower epigastric vessels or their branches during trocar placement and dissection in the TEPt group was brought under control with clip and suture ligation. No conversions occurred due to this bleeding. Eklund et al. reported bleeding from epigastric vessels in merely 1% of patients in their study [4].

The incidence of hematoma is reported at lower rates after TEPt compared to LSt repair. Most hematomas heal spontaneously in four weeks on average. However, large, symptomatic, or infected hematomas may require immediate surgical intervention [5]. In this study, one patient in the LSt group developed a hematoma. The patient was reoperated, but the cause of the bleeding could not be determined.

Isolated internal iliac artery aneurysms are rare, and only a few cases have been reported in the literature [12]. TEPt was applied to a patient with a history of left varicocelectomy diagnosed with bilateral IH. Pelvic hematoma developed in the early postoperative period in this case. Extravasation was seen on CT angiography, and the patient was treated with endovascular coil embolization with the diagnosis of pseudoaneurysm. In this case, the bleeding was caused by one of the terminal branches of the left internal iliac artery; however, this patient had to be reoperated after six months due to a recurrent right IH. The cause of recurrence was the dislocation and degradation of the mesh due to the hematoma.

In one study, the e-TEP technique was applied in 94 cases diagnosed with an inguinoscrotal hernia. Seroma requiring aspiration was reported in only one of six cases of large scrotal hernia [13]. Although there was no significant difference between the groups, symptomatic seroma was more common in the LSt than in the TEPt group. The seroma usually became apparent in the first 10 postoperative days and disappeared completely within 30-40 days. In some cases of symptomatic seroma, aspiration may be required. Although excessive dissection, insufficient hemostasis, and dead spaces are responsible for seroma formation, some studies suggest that it may occur due to the inflammatory response against polypropylene mesh [11,14,15].

In this study, to reduce the incidence of seroma, we applied some procedures such as fixing the hernia sac to Cooper's ligament or posterior abdominal wall with tacker or sutures, preperitoneal or scrotal drain placement, and cavity reduction with endo-loop. Hernia guidelines recommend interventions only in cases of symptomatic seromas lasting more than six weeks [1]. There was no significant difference between the groups regarding symptomatic and asymptomatic seroma in this study. Schmedt et al. have reported higher seroma rates in TEPt than in LSt repair in their meta-analysis (3.6-4.4% vs. 0.5-1.2%) [15].

PP due to peritoneal tear may cause conversion by restricting working space in the TEPt procedure. In one study, PP was reported in six (7.9%) patients in the TEPt group; however, only two (2.6%) of these cases were converted to LSt repair [16]. Langeveld et al. reported 21 (6.5%) conversions in their study. Two of these were converted to TAPP and the rest to LSt. In the same study, the major causes of conversion were reported to be adhesions (38%), peritoneal ruptures (14%), and bleeding [14].

Sigmoid colon (sliding type hernia) injury occurred in one recurrent case during sac dissection during the TEPt procedure. In this case, a 2-cm colon defect was observed, which was repaired by converting to the open technique. However, since mobilization of the sigmoid colon loop could not be achieved through the open anterior approach, mobilization of the colonic segment and closure of the peritoneum were completed with TAPP (hybrid technique).

Large peritoneal defects were closed by suture, hem-o-lok, endo-loop, or sealing by LigaSure instrument. Defects smaller than 10 mm that do not obstruct vision may not require closure. Although they do not restrict the working area, it is recommended that defects larger than 20 mm should be repaired due to the potential risk of intestinal or omentum incarceration [12,17].

Improper surgical technique is one of the most notable causes of recurrence after primary IH repair. Moreover, the structure and quality of the mesh and insufficient laying or fixation are other causes of recurrence [1]. One of the critical parameters to evaluate IH repair techniques is the rate of recurrence. Diverse risk factors have been identified in many studies in the literature, including surgeon-related issues (insufficient experience, improper technique), advanced age, gender, comorbidity, smoking, collagen diseases, obesity, ascites, previous pelvic surgery, pelvic radiation, recurrence cases, high ASA score, wrong size and mesh, insufficient fixation, and complex cases (irreducible, scrotal hernia, and emergency cases) [5,18].

One study reported significantly lower hernia recurrence rates after TEPt and LSt repairs performed by experienced surgeons (0.5% vs. 4.2%) [2]. It has been suggested that reoperation rates can be considered a measure of postoperative recurrence rates, given that recurrence is twice as common as reoperations [19]. Patients with postoperative early hematoma and those who undergo emergency surgery might be at risk of recurrence; however, a definitive relationship could not be established. Indeed, the occurrence of two recurrence cases in the TEPt group after postoperative hematomas supports this view [18].

In this study, two patients in the LSt group and two in the TEPt group were reoperated for recurrence. One of the cases involved a pseudoaneurysm that developed in the early postoperative period and underwent endovascular coil embolization. The other case involved recurrence after hematoma, which occurred in the postoperative period and healed spontaneously, like the previous case. There was no significant difference between the LSt and TEPt groups regarding recurrence-related reoperation rates. Large-scale Swedish, Danish, and German Herniamed Registry studies have reported reoperative recurrence rates of 0.61% vs. 1.08%, 3.3% vs. 2.4%, 0.8% vs. 1.0% for TEPt and LSt repair, respectively [5,8,20].

Chronic postoperative groin pain is a bothersome, moderate-intensity pain that lasts at least three months after surgery and adversely affects daily activities. Chronic persistent pain occurs in roughly 10-12% of patients who undergo IH surgery [1]. Avoiding excessive and deep dissection of the "triangle of pain" could be a solution for ilioinguinal or genitofemoral nerve injury in laparoscopic repair [21]. Chronic pain was more common in the LSt than in the TEPt group in this study; however, the difference was not statistically significant. The pain persisted in one patient in the LSt group for about seven months despite medical treatment and nerve block injections. The patient was reoperated, and it was observed that the mesh was extensively adherent to the ilioinguinal nerve. The mesh was extracted, and a neurectomy was performed.

Testicular complications are one of the notable complications of IH surgery. In this study, spermatic cord edema was more common in the LSt group. Testicular complications were not observed in our research, except for an incidence of planned orchiectomy in one patient with a scrotal hernia. Spermatic cord and testicular issues are associated with aggressive dissection and uncontrolled use of energy devices during the hernia sac dissection [1]. Acute testicular ischemia can be prevented by preserving the cremasteric vessels. The risk of ischemic orchitis increases in recurrent IH cases, especially when sac dissection is completely reduced in scrotal hernias; preservation of the cremasteric vessels and transection of the distal part of the hernia sac are recommended to reduce the risk of ischemic orchitis [22].

Ultimately, both LSt and TEPt can be safely performed in primary and complex cases with the proper approach, appropriate patient selection, and sufficient surgical experience. The surgeons must have enough experience with laparoscopic and open techniques to achieve favorable outcomes.

This study has a few limitations. This was a retrospective study and the sample size was low. On the other hand, the operations were performed by a specialist surgeon with open and laparoscopic experience, and the surgical data (including video recordings of the TEPt procedure) were meticulously recorded and analyzed, which are some of the vital and positive aspects of the present study.

## Conclusions

This study has revealed no significant difference between TEPt and LSt repair in terms of bleeding/hematoma, symptomatic seroma, chronic pain, and rate of recurrence. However, rather than speculating on the superiority of the two techniques, we believe it is more appropriate to recommend that the surgical technique should be chosen based on an analysis of risk factors depending on the patient and hernia type. However, more large-scale, high-quality, and long-term randomized controlled studies must be conducted to support this consideration.
